# New-onset supraventricular arrhythmia during septic shock: prevalence, risk factors and prognosis

**DOI:** 10.1186/s13613-015-0069-5

**Published:** 2015-09-22

**Authors:** Aurélien Seemann, Florence Boissier, Keyvan Razazi, Guillaume Carteaux, Nicolas de Prost, Christian Brun-Buisson, Armand Mekontso Dessap

**Affiliations:** AP-HP, CHU Henri Mondor, DHU A-TVB, Service de Réanimation Médicale, 51, avenue du Mal de Lattre de Tassigny, 94 010 Créteil Cedex, France; AP-HP, CHU Henri Mondor, DHU A-TVB, Service de Cardiologie, 94010 Créteil, France; AP-HP, Hôpital Européen Georges Pompidou, Service de Réanimation Médicale, 75015 Paris, France; Université Paris Est Créteil, Faculté de Médecine, Groupe de recherche clinique CARMAS, 94010 Créteil, France; INSERM U955, IMRB, Faculté de Médecine de Créteil, 94010 Créteil, France

**Keywords:** Arrhythmia, Sepsis, Shock, Myocardial dysfunction

## Abstract

**Background:**

The aims of this study were to prospectively assess the prevalence of sustained (lasting more than 30 s) new-onset supraventricular arrhythmia (NOSVA) during septic shock, identify the associated factors (including septic myocardial dysfunction), and evaluate its impact on hemodynamics and prognosis.

**Methods:**

Patients with a diagnosis of septic shock were screened in a medical intensive care unit of a tertiary hospital center in France with a continuous 12-lead EKG for the occurrence of NOSVA. Biological and clinical data (including septic myocardial dysfunction characterized by echocardiography) were collected. We also assessed the hemodynamic tolerance and prognosis of NOSVA.

**Results:**

Among the 71 septic shock episodes assessed during the study, NOSVA occurred in 30 [prevalence of 42 %, 95 % confidence interval (CI) 30–53 %]. Among all recorded factors, only renal failure (as assessed by renal SOFA score at day 1) was associated with NOSVA and this difference persisted by multivariable analysis (odds ratio of 1.29, 95 % CI 1.03–1.62, *p* = 0.03). There was a significant increase in norepinephrine dosage during the first hour after SVA onset. NOSVA was associated with longer catecholamine use during septic shock as compared with patients in sinus rhythm, whereas ICU mortality was identical between groups.

**Conclusions:**

We found a high prevalence of sustained NOSVA during septic shock. NOSVA was not related to septic myocardial dysfunction, but rather to acute renal failure, raising the hypothesis of an acute renocardiac syndrome.

## Background

Supraventricular arrhythmia (SVA) is the most common cardiac rhythm disturbance occurring in intensive care unit (ICU) patients [1]. Sepsis is frequently associated with new-onset SVA (NOSVA), which has been reported in 6 % of patients with severe sepsis [2] and in 46 % of those with septic shock [3]. Data evaluating risk factors for NOSVA during septic shock are scarce [2, 4].

Septic myocardial dysfunction is characterized by an acute, reversible depression of left ventricle (LV) contractility with normal or low filling pressures [5]. Its mechanisms are not fully understood, though circulating cytokines or cellular respiration alteration resulting from mitochondrial dysfunction may play a crucial role [6]. Septic cardiomyopathy may also involve diastolic dysfunction [7] and right ventricle dysfunction [8]. Whether septic cardiomyopathy may constitute an arrythmogenic condition is unknown to date. In addition, whether inotropic drugs used to correct LV systolic dysfunction in septic shock patients [8] may increase the risk of NOSVA remains unclear.

SVA has been associated with increased morbidity related to circulatory compromise, cardio-embolic events or hemorrhagic complications of anticoagulant treatments. Little is known about the prognostic impact of NOSVA in the context of septic shock [2].

The aim of our study was to prospectively assess the prevalence of NOSVA during septic shock, to identify the associated factors (including the role of septic myocardial dysfunction), and to evaluate its impact on hemodynamics and outcome of patients.

## Patients and methods

### Patients

Patients who met septic shock criteria (as defined according to the ACCP/SCCM Consensus Conference [9]) were prospectively included at the medical ICU of Henri Mondor University Hospital (Créteil, France) between October 2011 and December 2012. Non-inclusion criteria were cardiac surgery within the preceding month, chronic heart failure (defined by a baseline left ventricular ejection fraction below 45 %) or a history of persistent SVA. The study was approved by the institutional ethics committee (Comité de Protection de Personnes Ile de France IX) as a component of standard care and patient’s consent was waived. Written and oral information about the study was given to the families. Patient’s severity was evaluated by the McCabe and Jackson score for underlying diseases [10], the SAPS II score for acute illness at ICU admission [11] and the SOFA (sequential organ failure assessment) score for organ dysfunction during septic shock [12]. Chronic renal failure was defined as a glomerular filtration rate below 30 ml/min/1.73 m^2^ according to MDRD formula [13]. Acute renal failure and other organ failures were defined using the SOFA score [12]. Acute respiratory distress syndrome (ARDS) was defined according to the Berlin criteria [14]. Blood concentration of thyroid stimulating hormone, NT-pro BNP and cardiac troponin T were assessed using an immuno-assay on a COBAS 6000 analyzer (Roche Diagnostics, Boulogne-Billancourt, France). Follow-up for the study was at least until ICU discharge.

### NOSVA

All patients were continuously monitored with a reconstructed 12-lead EKG (using a continuous 6-lead cardiac monitoring, Dräger Infinity^®^ Acute Care System, Antony, France). EKG data were automatically recorded, digitally stored and checked daily for occurrence of NOSVA during the entire course of septic shock. SVA episodes lasting more than 30 s were assessed by two cardiologists (any discrepancy being solved by consensus) and classified as atrial fibrillation, atrial flutter or atypical flutter, using standard definitions [15].

### Septic myocardial dysfunction

To evaluate cardiac function, we used transthoracic echocardiography (TTE) or multiplane transesophageal echocardiography (TEE), when TTE did not allow accurate measurements because of poor acoustic windows. Echocardiograms were performed by trained operators (competence in advanced critical care echocardiography) [16] using an iE33 system (Philips Ultrasound, Bothell, WA, USA) with a standard procedure [17]. Briefly, the following echocardiographic views were examined: four-chamber and two-chamber long-axis views to assess left ventricle (LV) ejection fraction (computed from LV volumes using the bi-plane Simpson method [18] when image quality was good, or visually estimated when poor image quality did not allow sufficient identification of the endocardium [19]), right and left atrium size [18], right ventricle size (a dilated RV was defined by an end-diastolic RV/LV area ratio >0.6) [20]), right ventricle function (using the tricuspid annular plane systolic excursion and tissue Doppler peak systolic wave at the tricuspid valve annulus [21]); long-axis M-mode view of the superior (TEE) or inferior (TTE) vena cava to assess their respiratory variability [22]; and the presence of pericardial effusion. Pulsed-wave Doppler aortic flow was obtained at the level of the aortic annulus and the velocity–time integral was automatically processed by tracing the envelope of aortic flow for cardiac index calculation. Echocardiographic images were digitally stored, and a computer-assisted evaluation was performed off-line by two trained operators. Septic cardiac dysfunction was defined as an LVEF <45 % or the need for an inotrope infusion in order to achieve an LVEF ≥45 %.

### Statistical analysis

The data were analyzed using SPSS Base 13.0 (SPSS Inc, Chicago, IL, USA) and R 2.15.2 (The R Foundation for Statistical Computing, Vienna, Austria) statistical software packages. Continuous data were expressed as median [25th–75th percentiles], unless otherwise specified and were compared using the Mann–Whitney test for independent samples and the Friedman test for related samples. Categorical variables, expressed as percentages, were evaluated using the Chi-square test or Fisher exact test. To evaluate independent factors associated with NOSVA (time-dependent variable), significant or marginally significant univariate risk factors (*p* < 0.10) recorded prior or at the time of the first episode of NOSVA, were examined using backward stepwise multivariate Cox proportional-hazards regression model; septic myocardial dysfunction and patient severity as assessed by SAPS II score at ICU admission were also included in the model; thus, the five variables included in the model were SAPS II score at ICU admission, age, septic myocardial dysfunction, renal and non-renal SOFA scores at day 1 of septic shock. Coefficients were computed by the method of maximum likelihood. Two-tailed *p* values lower than 0.05 were considered significant.

## Results

### Patients’ characteristics and NOSVA

Eighty-six patients were admitted in our unit with a diagnosis of septic shock during the study period. Among them, 21 patients were excluded because of cardiac surgery within the preceding month (*n* = 8), chronic heart failure (*n* = 6), history of permanent atrial fibrillation (*n* = 4) or logistic difficulties impeding proper data collection (*n* = 3). Thus, a total of 65 patients were included in the study, with 71 episodes of septic shock (six patients exhibited two distinct episodes of septic shock during the study period). Baseline characteristics of included patients are displayed in Table 1. SAPS II at ICU admission was 51 [39–68], and SOFA score at onset of septic shock was 10 [7–14]. NOSVA occurred during 30 septic shock episodes (in 27 patients), defining a prevalence of 42 % (95 % CI 30–54 %). In the six patients with two distinct episodes of septic shock (more than 1 week apart), three experienced a paroxysmal NOSVA during the first episode with a relapse during the second episode, one experienced NOSVA during the first episode only, and two remained in sinus rhythm during both episodes. NOSVA subtypes were atrial fibrillation (*n* = 23), atypical flutter (*n* = 4) and atrial flutter (*n* = 3). The median time from septic shock onset to NOSVA was 2 [1–4] days, with 15 (50.0 %) episodes occurring on the first day of septic shock. NOSVA occurred only once during 19 septic shock episodes and occurred two or more times during 11 septic shock episodes. The median cumulative length of NOSVA was 19.5 [3–57] h.

**Table 1:** Patients’ baseline characteristics, according to the occurrence of new-onset supraventricular arrhythmia during at least one septic shock episode

	NOSVA during septic shock episode		*p* value
	Never (*n* = 38)	Ever (*n* = 27)	
Age, years	55.5 [48.5–68.5]	66 [56–75.0]	0.071
Weight, kg	73.0 [57.0–85.0]	72.0 [64.0–89.0]	0.464
Height, cm	171 [164–178]	170.0 [162.55–173.5]	0.468
Female sex	15 (39.5)	14 (51.9)	0.323
History of cardiac disease	15 (39.5)	6 (22.2)	0.143
Coronary artery disease	7 (18.4)	4 (14.8)	0.751
Paroxysmal SVA	3 (7.9)	5 (18.5)	0.260
Valvular heart disease	3 (7.9)	1 (3.7)	0.636
PM or ICD	2 (5.3)	1 (3.7)	>0.99
Chronic renal failure	3 (8.1)	2 (7.4)	>0.99
Chronic dialysis	2 (5.3)	2 (7.4)	>0.99
History of stroke	3 (7.9)	3 (11.1)	0.686
History of thyroid dysfunction	2 (5.3)	1 (3.7)	>0.99
Diabetes	8 (21.1)	5 (18.5)	0.801
Smoker	11 (28.9)	8 (29.6)	0.952
History of dyslipidemia	5 (13.2)	5 (18.5)	0.729
History of hypertension	15 (39.5)	8 (29.6)	0.413
Betablocker use	13 (34.2)	7 (25.9)	0.589
Amiodarone use	4 (10.5)	2 (7.4)	>0.99
McCabe score	1.0 [0.0–2.0]	1.0 [0.0–2.0]	0.321
SAPS II score at ICU admission	48.5 [33.2–62.2]	56 [40–71]	0.136

Data are *n* (%) or median [25th–75th percentile] unless otherwise specified

Chronic renal failure was defined as a glomerular filtration rate below 30 ml/min/1.73 m^2^ according to simplified MDRD formula [13]

*NOSVA* new-onset supraventricular arrhythmia, *PM* pacemaker, *ICD* intra-cardiac defibrillator, *SAPS* Simplified Acute Physiologic Score, *ICU* intensive care unit, *LVEF* left ventricular ejection fraction

### Factors associated with NOSVA occurrence

There was no significant difference in baseline characteristics between patients with or without NOSVA, including cardiovascular risk factors, cardiac medications, chronic renal failure, thyroid dysfunction, and SAPS II score at ICU admission (Table 1). Organ dysfunction and treatments administered during septic shock according to the occurrence of NOSVA are displayed in Table 2. Renal SOFA score at day 1 of septic shock was significantly higher in patients with NOSVA. Among the 11 patients with a NOSVA episode who needed dialysis during shock, arrhythmia occurred before dialysis initiation in six patients. Other organ dysfunctions (neurological, cardiovascular, hematological, respiratory, coagulation and hepatic) had similar rates between groups. Daily fluid balance was not associated with the occurrence of NOSVA. Maximal doses of catecholamines infused did not differ between patients with NOSVA and others. Biological data, including blood potassium, thyroid stimulating hormone, cardiac Troponin T and NT-pro-BNP levels were similar between groups. Respiratory variables and the use of superior vena cava central line were similar between groups. Overall, steroid use for septic shock was significantly higher among patients with NOSVA as compared to those without, but this difference disappeared when considering only steroids administered before SVA onset.

**Table 2:** Organ dysfunction, biological data and treatments during septic shock according to supraventricular arrhythmia occurrence

	Sinus rhythm (*n* = 41)	SVA (*n* = 30)	*p*
SOFA day 1			
Global (0–24)	10.0 [7.0–12.5]	10 [8–16]	0.369
Neurological (0–4)	1.0 [0.0–4.0]	1.0 [0.0–4.0]	0.869
Respiratory (0–4)	2.0 [0.0–4.0]	2.0 [0.8–3.0]	0.976
Cardiovascular (0–4)	4.0 [4.0–4.0]	4.0 [4.0–4.0]	0.709
Renal (0–4)	1.0 [0.0–2.5]	2.0 [1.0–4.0]	0.034
Coagulation (0–4)	0.0 [0.0–1.5]	1.0 [0.0–2.0]	0.573
Hepatic (0–4)	0.0 [0.0–2.0]	0.0 [0.0–1.25]	0.875
Day 1 blood gases			
pH	7.33 [7.21–7.41]	7.32 [7.24–7.40]	0.802
*P*CO_2_, mmHg	37.0 [29.0–44.5]	36.0 [30.7–47.5]	0.692
*P*O_2_, mmHg	123.0 [84.5–185.5]	102.0 [76.0–215.2]	0.802
Bicarbonates, mmol/L	19.0 [15.9–25.2]	20.5 [17.0–25.0]	0.518
Lactate, mmol/L	2.90 [1.43–4.30]	2.10 [1.40–3.60]	0.514
FIO_2_, %	100 [55–100]	100 [70–100]	0.910
PEEP, cmH_2_O	5 [5]	5 [5]	0.759
Thyroid stimulating hormone, µUI/L	0.94 [0.35–3.12]	1.04 [0.34–2.26]	0.694
Cardiac Troponin peak between day 1 and day 3, ng/L	44 [12.5–295]	157.5 [22.7–545.8]	0.222
NT proBNP peak between day 1 and day 3, pg/mL	8092 [2424–32,410]	15,522 [5987–46209]	0.229
Minimal potassium level during septic shock, mmol/L	3.3 [2.8–3.7]	3.2 [2.9–3.4]	0.571
Maximal potassium level during septic shock, mmol/L	4.6 [4.3–5.2]	4.5 [4.2–5.1]	0.771
Hemodynamic treatments			
Cumulative fluid balance during shock, mL	5358 [2401–9965]	4233 [3241–9682]	0.979
Daily fluid balance during shock, mL/day	2285 [1554–3825]	1387 [1080–1859]	0.106
Dobutamine use during shock	8.0 (19.5)	5 (16.7)	0.759
Dobutamine maximal dose, µg/kg/min^a^	5.0 [5.0–13.8]	10.0 [5.0–12.5]	0.622
Dobutamine maximal dose, µg/kg/min, mean (SD)^a^	7.8 (4.9)	9.0 (4.2)	
Norepinephrine use during shock	40 (97.6)	29 (96.7)	>0.99
Norepinephrine maximal dose, mg/h^a^	2.9 [1.2–8.0]	3.3 [2.0–10.0]	0.210
Norepinephrine maximal dose, mg/h, mean (SD)^a^	5.1 (5.5)	6.7 (6.4)	
Epinephrine use during shock	3 (7.3)	3 (10.0)	0.692
Dual catecholamine use during shock	10 (24.4)	6 (20.0)	0.662
Length of catecholamine use during shock, days	3 [2–5]	4 [3–7]	0.035
Length of vasopressor use during shock, days	3 [2–5]	4 [3–7.0]	0.021
Respiratory treatments			
Invasive mechanical ventilation	33 (80.5)	27 (90.0)	0.335
Mild ARDS	7 (17.9)	7 (24.1)	0.532
Moderate to severe ARDS	17 (41.5)	11 (36.7)	0.683
Other treatments			
Dialysis during shock	7 (17.1)	11 (36.7)	0.061
Steroid use during shock	25 (61.0)	25 (83.3)	0.041
Steroid use before SVA onset	25 (61.0)	14 (46.7)	0.231
Superior vena cava central line in place during shock	13 (31.7)	10 (33.3)	0.885

Data are *n* (%) or median [25th–75th percentile] unless otherwise specified

*SVA* supraventricular arrhythmia, *SOFA* sepsis-related organ failure assessment, *PEEP* positive end-expiratory pressure, *ARDS* acute respiratory distress syndrome

^a^Only patients receiving the drug during septic shock were considered

Temperature and biological data at the time of the first occurrence of SVA are displayed in Table 3. Overall, these variables were within the normal range in the majority of patients experiencing the first SVA episode, except for elevated serum urea and creatinine which were common in this group.

**Table 3:** Body temperature and biological variables stratified according to their reference limits, at time of first occurrence of supraventricular arrhythmia during septic shock

Variables	SVA (*n* = 30)
Temperature, °C	37.3 [36.0–38.2]
Temperature >38.3 °C	7 (21.9)
Temperature <36.0 °C	4 (12.5)
Potassium level, mmol/L	3.7 [3.4–4.2]
Potassium level <3.5 mmol/L	8 (25.0)
Potassium level >5 mmol/L	2 (6.3)
Glycemia, mmol/L	6.5 [5.5–8.4]
Glycemia <4 mmol/L	3 (9.7)
Glycemia >10 mmol/L	6 (19.4)
pH	7.39 [7.29–7.44]
pH <7.38	14 (43.8)
PaCO_2_, mmHg	34.0 [30.0–40.2]
PaO_2_, mmHg	100 [85–164]
PaO_2_ <60 mmHg	1 (3.1 %)
SaO_2_ (%)	98 [96–100]
Bicarbonates, mmol/L	21.2 [18.0–24.8]
Lactates, mmol/L	1.6 [1.3–3.3]
Serum creatinine, µmol/L	157 [90–272]
Serum creatinine >130 µmol/L	20 (62.5)
Serum urea, mmol/L	12.9 [7.9–21.1]
Serum urea >7 mmol/L	26 (81.3)
PaO_2_/FiO_2_ ratio	228 [175–340]

Data are *n* (%) or median [25th–75th percentile] unless otherwise specified

*SVA* supraventricular arrhythmia

Table 4 shows echocardiographic parameters in patients with and without NOSVA. As expected, echocardiograms were performed less often in sinus rhythm in the NOSVA group. Cardiac chamber sizes, presence of pericardial effusion as well as median LVEF were similar between the two groups. The presence of septic myocardial dysfunction was similar between groups (*p* = 0.303 and *p* = 0.329 for Chi-square test and log-rank test, respectively).

**Table 4:** Echocardiographic data during septic shock

	Sinus rhythm (*n* = 41)	SVA (*n* = 30)	*p*
Echocardiogram on sinus rhythm	40 (97.6)^a^	23 (76.7)	0.006
Echocardiogram under inotropes	2 (4.9)	4 (13.3)	0.233
LVEF, %	60 [44–60]	59 [44–60]	0.587
Septic myocardial dysfunction (*n* = 69)^b^	11 (28.2)	12 (40.0)	0.303
Cardiac index, L/min/m^2^	2.8 [2.2–3.6]	3.4 [2.5–3.8]	0.131
*E*/*e*′ ratio	8.5 [6.9–11.5]	8.3 [5.3–10.8]	0.547
RV/LV ratio	0.6 [0.4–0.7]	0.5 [0.4–0.7]	0.818
RV dilatation	20 (51.3)	12 (42.9)	0.496
RA size, cm^2^	12.1 [11.0–17.5]	15.6 [11.0–19.7]	0.422
LA size, cm^2^	17.0 [14.2–21.0]	18.0 [14.0–19.5]	0.879
TAPSE, mm	20.0 [17.0–22.0]	17.0 [13.8–25.0]	0.150
Tricuspid tissue Doppler s’ wave, cm/s	13 [10–14]	13 [8–15]	0.812
Pericardial effusion	3 (7.3)	4 (13.3)	0.446

Data are *n* (%) or median [25th–75th percentile]

*SVA* supraventricular arrhythmia; *E/e*′ *ratio* ratio of transmitral Doppler early (*E*) filling velocity to tissue Doppler early diastolic mitral annular velocity; *RA* right atria; *LA* left atria; *LV* left ventricle; *RV* right ventricle, RV dilatation was defined as end-diastolic area ratio >0.6; *TAPSE* tricuspid annular plane systolic excursion

^a^One patient had internal pacing

^b^Two patients were excluded from this analysis because of a poor echogenicity

In the Cox proportional-hazards regression analysis adjusted for septic myocardial dysfunction, SAPS II score and renal and non-renal SOFA scores at day 1 of septic shock, the only factor significantly associated with NOSVA was the renal SOFA score at day 1 of septic shock (hazard ratio of 1.29, 95 % CI 1.03–1.62, *p* = 0.03; Table 5).

**Table 5:** Factors associated with new-onset supraventricular arrhythmia occurrence by Cox analysis

Variable	Hazard ratio (95 % CI), *p* value	
	Univariate	Multivariable
SAPS II score at ICU admission	1.02 (1.00–1.03), *p* = 0.05	I/NR
Age	1.02 (0.99–1.05), *p* = 0.23	I/NR
Septic myocardial dysfunction	1.40 (0.68–2.92), *p* = 0.36	I/NR
Renal SOFA	1.28 (1.02–1.60), *p* = 0.03	1.29 (1.03 1.62), *p* = 0.03
Non-renal SOFA	0.99 (0.90–1.11), *p* = 0.97	I/NR

*SAPS* Simplified Acute Physiologic Score; *SOFA* sepsis-related organ failure assessment; *ICU* intensive care unit; *I/NR* included, but not retained in the final model

### Treatment of NOSVA

A treatment for cardioversion was administered in 21 of 30 episodes of NOSVA, including magnesium sulfate (*n* = 6), amiodarone (*n* = 19), and/or electric shock (*n* = 3). Cardioversion was effective in 18 (86 %) cases. Curative anticoagulant treatment with heparin was used in eight (27 %) patients with NOSVA; in the other cases, the attending physician considered that the benefit/risk ratio of curative anticoagulation was unfavorable. No stroke was diagnosed among patients with NOSVA up to ICU discharge.

### Outcome of NOSVA

Table 6 shows hemodynamic changes induced by the first recorded episode of NOSVA. There was a trend towards a decrease in mean, systolic and pulse arterial pressure despite a significant increase in norepinephrine dosage during the first hour after SVA onset. NOSVA was associated with longer catecholamine use and longer vasopressor use during septic shock (Table 2). ICU mortality was identical between patients with NOSVA and those without [16 (42.1 %) vs. 13 (48.1 %), *p* = 0.63], whereas there was a trend towards prolonged ICU length of stay in ICU survivors in the former group (16.0 [10.5–32.0] days vs. 8.5 [4.0–24.5] days, *p* = 0.08).

**Table 6:** Hemodynamic data immediately before and during the first hour of the first episode of supraventricular arrhythmia

	Before SVA onset (*n* = 30)	After SVA onset (*n* = 30)	*p*
Systolic arterial pressure, mmHg	114 [97–127]	97 [86–118]	0.076
Diastolic arterial pressure, mmHg	58 [51–63]	58 [47–64]	0.513
Mean arterial pressure, mmHg	77.0 [70.0–87.0]	73.0 [58.5–80.5]	0.056
Arterial pulse pressure, mmHg	54 [39–70]	42 [31–57]	0.081
Heart rate, beats/min	99 [87–118]	140 [123–165]	<0.001
Dobutamine dose, µg/kg/min^a^	7.5 [5.0–10.0]	7.5 [5.0–10.0]	>0.99
Norepinephrine dose, mg/h^a^	1.7 [0.3–3.0]	1.8 [0.5–4.5]	0.010
Epinephrine dose, mg/h^a^	1.3 [0.5–2.0]	1.5 [0.0–3.0]	0.655

Data are n (%) or median [25th–75th percentile] unless otherwise specified

*SVA* supraventricular arrhythmia

^a^Only patients receiving the drug before or after SVA onset were considered

### Sensitivity analyses

When repeating the analyses while considering only the first episode of septic shock in patients with multiple episodes, we found concordant results: (1) in the Cox proportional-hazards regression analysis, the only factor associated with NOSVA was the renal SOFA score at day 1 of septic shock [hazard ratio of 1.26 (95 % CI 0.99–1.60), with a marginally significant *p* value (*p* = 0.06]; (2) NOSVA was associated with longer catecholamine use (4 [3–7] vs. 3 [2–6], *p* = 0.049), and vasopressor use (4 [3–7] vs. 3 [2–5], *p* = 0.03) during septic shock, and a trend towards longer ICU length of stay in survivors (16.0 [10.0–32.0] days vs. 8.5 [4.0–24.0] days, *p* = 0.067).

## Discussion

We report a high prevalence of NOSVA (42 %; 95 % CI [30–54 %]) during septic shock. NOSVA was not associated with septic myocardial dysfunction, but with renal failure by multivariable analysis. Patients with NOSVA exhibited longer durations of shock and a trend towards longer ICU stay.

The prevalence of NOSVA in our study is higher than reported in the general ICU population [1, 2, 23], but comparable to the 46 % atrial fibrillation rate reported by Meierhenrich et al. [3] in a cohort of patients with septic shock. The higher prevalence of SVA in septic shock patients might be related to their higher severity of illness. The use of inotropes in septic shock patients is unlikely to fully explain such a difference, since many of our patients presented NOSVA before initiating catecholamine support.

We were unable to demonstrate an association between the occurrence of NOSVA and septic myocardial dysfunction, as assessed by LVEF. Several explanations are possible. First, LVEF may not be the ideal criterion to assess septic cardiomyopathy, notably because of its load-dependency. However, evaluation of cardiac function in our patients was done after fluid loading and while receiving vasoconstrictors. Whether echocardiographic parameters less dependent on afterload, such as speckle tracking derived strain-rate, may better characterize septic cardiomyopathy warrants further research. Second, the mechanisms of septic myocardial dysfunction and substrates/triggers of SVA may differ. Septic cardiomyopathy mainly affects heart ventricles, with depressed contractility and low or normal filling pressures [6], whereas SVA is an atrial disorder that is mainly influenced by atrial pressure and autonomous nervous system tone. Cardiac biomarkers (B-type natriuretic peptides and troponins), which are mainly released from the ventricular myocytes and mostly influenced by left ventricle function and mass, were not significantly associated with SVA in our series despite a trend towards higher values in the NOSVA group.

We found an association between NOSVA and renal failure by univariate and multivariable analysis. Patients with NOSVA exhibited higher values of serum urea and creatinine, and a higher renal SOFA score as compared to patients in sinus rhythm, whereas the prevalence of chronic renal failure and chronic dialysis was similar between groups. Cardiorenal syndrome (CRS) is a complex pathophysiological disorder of the heart and kidneys in which acute or chronic dysfunction in one organ may induce acute or chronic dysfunction in the other organ [24]. The association we observed between acute renal failure and NOSVA may correspond to either a type 1 CRS (acute cardiorenal syndrome, i.e., an acute deterioration in cardiac function that leads to acute kidney injury), a type 3 CRS (acute renocardiac syndrome, i.e., an acute kidney injury that leads to acute cardiac injury), or a type 5 CRS (secondary cardiorenal syndrome, i.e., a systemic disorder causing cardiac and renal dysfunction) [25, 26]. Hemodynamic alteration during SVA episodes might have impeded renal perfusion, thus leading to an acute cardiorenal syndrome. However, the renal SOFA score was significantly different between the NOSVA group and the sinus rhythm group from the very start of septic shock (day 1), favoring the hypothesis of an acute renocardiac syndrome. The mechanisms underlying acute renocardiac syndrome are not clearly understood, but may involve direct (e.g., cytokines, sympathetic tonus and renin–angiotensin–aldosterone system) or indirect (e.g., fluid and electrolyte imbalances) effects of acute kidney injury on the heart [27, 28]. However, in our study, the potential role of dyskalemia and fluid balance on the occurrence of NOSVA did not seem pivotal. Further studies are needed to better scrutinize the spectrum of acute renocardiac syndrome in septic shock patients.

As expected, the occurrence of NOSVA during septic shock was associated with a poor acute hemodynamic tolerance, with a fall in arterial pressure and increased doses of catecholamines. Indeed, the loss of auricular systole and tachycardia-related shortening of ventricular diastole may both contribute to ventricular filling impairment. NOSVA was also associated with longer use of drugs and vasopressors, and a trend towards longer ICU stay. Whether NOSVA is a direct cause of prolonged catecholamine use and longer ICU stay or merely an indicator of severity of illness remains to be determined. The absence of clinical ischemic stroke in our study despite a low curative anticoagulation rate underscores the need to weight the benefit/risk ratio of this treatment for each patient with NOSVA in the ICU setting.

Our study has several limitations. First, the study power was limited by the inclusion of only 71 episodes of septic shock. We cannot formally exclude a possible relationship between septic myocardial dysfunction and NOSVA in a larger sample size. In view of the 30 events observed, we did not strictly follow the classical rule of a minimum of 10 outcome events per predictor variable in the multivariable analysis [29], but our analysis fulfilled the minimum of 5 events per predictor variable proposed by recent simulation studies [30]. Second, we included some patients with multiple septic shock episodes (more than 1 week apart). Our sensitivity analysis considering only the first episode yielded nearly similar results despite a reduced power. However, we cannot exclude a role for personal susceptibility in the occurrence of NOSVA. Third, we could not perform consecutive echocardiograms during sinus rhythm and at the onset of SVA to better scrutinize hemodynamic changes induced by SVA.

## Conclusions

In conclusion, our study showed that sustained NOSVA is frequent during septic shock, poorly tolerated, and is associated with longer catecholamine use. Renal failure, but not myocardial dysfunction, was significantly associated with occurrence of NOSVA during septic shock, raising the hypothesis of an acute renocardiac syndrome.

## References

[1] Annane D, Sebille V, Duboc D, Le Heuzey JY, Sadoul N, Bouvier E, Bellissant E (2008). Incidence and prognosis of sustained arrhythmias in critically ill patients. Am J Respir Crit Care Med.

[2] Walkey AJ, Wiener RS, Ghobrial JM, Curtis LH, Benjamin EJ (2011). Incident stroke and mortality associated with new-onset atrial fibrillation in patients hospitalized with severe sepsis. JAMA.

[3] Meierhenrich R, Steinhilber E, Eggermann C, Weiss M, Voglic S, Bogelein D, Gauss A, Georgieff M, Stahl W (2010). Incidence and prognostic impact of new-onset atrial fibrillation in patients with septic shock: a prospective observational study. Crit Care.

[4] Goodman S, Shirov T, Weissman C (2007). Supraventricular arrhythmias in intensive care unit patients: short and long-term consequences. Anesth Analg.

[5] Parker MM, Shelhamer JH, Bacharach SL, Green MV, Natanson C, Frederick TM, Damske BA, Parrillo JE (1984). Profound but reversible myocardial depression in patients with septic shock. Ann Intern Med.

[6] Vieillard-Baron A (2011). Septic cardiomyopathy. Ann Intensive Care.

[7] Bouhemad B, Nicolas-Robin A, Arbelot C, Arthaud M, Feger F, Rouby JJ (2009). Acute left ventricular dilatation and shock-induced myocardial dysfunction. Crit Care Med.

[8] Vieillard-Baron A, Caille V, Charron C, Belliard G, Page B, Jardin F (2008). Actual incidence of global left ventricular hypokinesia in adult septic shock. Crit Care Med.

[9] Levy MM, Fink MP, Marshall JC, Abraham E, Angus D, Cook D, Cohen J, Opal SM, Vincent JL, Ramsay G (2003). 2001 SCCM/ESICM/ACCP/ATS/SIS International Sepsis Definitions Conference. Crit Care Med.

[10] McCabe WR, Jackson GG (1963). Gram-negative bacteremia. Etiology and ecology. Arch Inern Med.

[11] Le Gall JR, Lemeshow S, Saulnier F (1993). A new Simplified Acute Physiology Score (SAPS II) based on a European/North American multicenter study. JAMA.

[12] Vincent JL, de Mendonca A, Cantraine F, Moreno R, Takala J, Suter PM, Sprung CL, Colardyn F, Blecher S (1998). Use of the SOFA score to assess the incidence of organ dysfunction/failure in intensive care units: results of a multicenter, prospective study. Working group on “sepsis-related problems” of the European Society of Intensive Care Medicine. Crit Care Med.

[13] Levey AS, Bosch JP, Lewis JB, Greene T, Rogers N, Roth D (1999). A more accurate method to estimate glomerular filtration rate from serum creatinine: a new prediction equation. Modification of Diet in Renal Disease Study Group. Ann Intern Med.

[14] Ranieri VM, Rubenfeld GD, Thompson BT, Ferguson ND, Caldwell E, Fan E, Camporota L, Slutsky AS (2012). Acute respiratory distress syndrome: the Berlin Definition. JAMA.

[15] Miller JMZD. Diagnosis of cardiac arrhythmias. In: Braunwald’s Heart Disease: a textbook of cardiovascular medicine, 9th edn; 2012. p. 687–702.

[16] Mayo PH, Beaulieu Y, Doelken P, Feller-Kopman D, Harrod C, Kaplan A, Oropello J, Vieillard-Baron A, Axler O, Lichtenstein D (2009). American College of Chest Physicians/La Societe de Reanimation de Langue Francaise statement on competence in critical care ultrasonography. Chest.

[17] Vieillard-Baron A, Prin S, Chergui K, Dubourg O, Jardin F (2003). Hemodynamic instability in sepsis: bedside assessment by Doppler echocardiography. Am J Respir Crit Care Med.

[18] Lang RM, Bierig M, Devereux RB, Flachskampf FA, Foster E, Pellikka PA, Picard MH, Roman MJ, Seward J, Shanewise JS (2005). Recommendations for chamber quantification: a report from the American Society of Echocardiography’s Guidelines and Standards Committee and the Chamber Quantification Writing Group, developed in conjunction with the European Association of Echocardiography, a branch of the European Society of Cardiology. J Am Soc Echocardiogr.

[19] Gudmundsson P, Rydberg E, Winter R, Willenheimer R (2005). Visually estimated left ventricular ejection fraction by echocardiography is closely correlated with formal quantitative methods. Int J Cardiol.

[20] Jardin F, Dubourg O, Bourdarias JP (1997). Echocardiographic pattern of acute cor pulmonale. Chest.

[21] Rudski LG, Lai WW, Afilalo J, Hua L, Handschumacher MD, Chandrasekaran K, Solomon SD, Louie EK, Schiller NB (2010). Guidelines for the echocardiographic assessment of the right heart in adults: a report from the American Society of Echocardiography endorsed by the European Association of Echocardiography, a registered branch of the European Society of Cardiology, and the Canadian Society of Echocardiography. J Am Soc Echocardiogr.

[22] Jardin F, Vieillard-Baron A (2006). Ultrasonographic examination of the venae cavae. Intensive Care Med.

[23] Christian SA, Schorr C, Ferchau L, Jarbrink ME, Parrillo JE, Gerber DR (2008). Clinical characteristics and outcomes of septic patients with new-onset atrial fibrillation. J Crit Care.

[24] Cruz DN (2013). Cardiorenal syndrome in critical care: the acute cardiorenal and renocardiac syndromes. Adv Chronic Kidney Dis.

[25] House AA, Anand I, Bellomo R, Cruz D, Bobek I, Anker SD, Aspromonte N, Bagshaw S, Berl T, Daliento L (2010). Definition and classification of Cardio-Renal Syndromes: workgroup statements from the 7th ADQI Consensus Conference. Nephrol Dial Transpl.

[26] Ronco C (2011). Cardio-renal syndromes: from foggy bottoms to sunny hills. Heart Fail Rev.

[27] Ronco C, Haapio M, House AA, Anavekar N, Bellomo R (2008). Cardiorenal syndrome. J Am Coll Cardiol.

[28] Chuasuwan A, Kellum JA (2012). Cardio-renal syndrome type 3: epidemiology, pathophysiology, and treatment. Semin Nephrol.

[29] Peduzzi P, Concato J, Kemper E, Holford TR, Feinstein AR (1996). A simulation study of the number of events per variable in logistic regression analysis. J Clin Epidemiol.

[30] Vittinghoff E, McCulloch CE (2007). Relaxing the rule of ten events per variable in logistic and Cox regression. Am J Epidemiol.

